# The impact of multi-site musculoskeletal pain on work ability among health care providers

**DOI:** 10.1186/s12995-015-0063-8

**Published:** 2015-05-27

**Authors:** Chanwit Phongamwong, Hemwarun Deema

**Affiliations:** Department of Rehabilitation Medicine, Phramongkutklao Hospital and Phramongkutklao College of Medicine, Bangkok, Thailand

**Keywords:** Multi-site pain, Number of pain sites, Musculoskeletal pain, Work ability, Health care providers

## Abstract

**Background:**

Epidemiologic studies have reported that multi-site musculoskeletal pain threatens work ability. However, no study has been conducted on this topic among health care providers. The aim of the present study was to determine the association between multi-site pain and poor work ability among health care providers.

**Methods:**

A cross-sectional study was conducted. Participants completed a self-administered questionnaire including basic characteristics, job satisfaction, stress screening, musculoskeletal pain at neck, upper extremities, low back, and lower extremities within the last month, and work ability index. Pain intensity was dichotomized according to a numerical pain rating scale score: less than five (no) and at least five (yes). Musculoskeletal pain was divided in three groups: 1) no pain, 2) few pain sites (one to two sites), and 3) many pain sites (three to four sites). The association of the number of pain sites with poor work ability was explored through multivariable logistic regression analysis.

**Results:**

A total of 254 health care providers participated in the present study. The majority of participants were female (73.2 %) with mean age of 33.9 (SD 9.5) years. Few pain sites and many pain sites were reported by 79 (31.1 %) and 39 participants (15.4 %), respectively. The adjusted odds ratio for poor work ability of participants who had few pain sites and many pain sites were 1.85 (95 % CI: 0.91 – 3.76) and 2.41 (95 % CI: 1.04 – 5.58), respectively.

**Conclusion:**

The present study showed that multi-site musculoskeletal pain had an association with poor work ability. The magnitude of association was likely to increase by a higher number of pain sites.

## Introduction

Currently, musculoskeletal pain has become a major health problem around the world [1]. The most recent report of the global burden caused by the 25 leading diseases and injuries in 2010 showed that low back pain ranked as the seventh with 80,667,000 of disability-adjusted life-years (DALYs), followed by neck pain as the twenty-first with 32,651,000 of DALYs. Other musculoskeletal disorders was ranked the twenty-third with 30,877,000 of DALYs, whereas ischemic heart disease ranked as the first with 129,795,000 of DALYs [2].

In the recent decade, many epidemiologic studies focused on multi-site musculoskeletal pain, and their findings revealed that the prevalence of multi-site pain is high in both general and working populations including hospital workers [3–11]. The prevalence among health care providers is about 40 – 50 % depending on definition of pain site and study population [4, 6].

Epidemiologic studies found the multi-site pain associates with poor work ability in working populations [12–14]. A prospective study among food-industrial workers demonstrated that the number of pain sites predicted poor work ability after 4 years of follow-up with a dose–response manner [13]. However, no epidemiologic evidence is available among the hospital working population. Hence, the aim of the present study was to confirm whether the number of pain sites has an association with poor work ability among health care providers.

## Methods

### Study design and participants

The present study was cross-sectional study conducted among health care providers of a tertiary hospital in Thailand between April 2014 and June 2014. The self-administered paper questionnaires were distributed in the hospital with convenient sampling. There was no reminder to complete the questionnaires. Eligibility criteria included being a health care provider including physicians, nurses, pharmacists, dentists, medical technicians, and physical/occupational therapists who worked in the hospital at least six months, were able to communicate in Thai, and consented to participate in the present study. Retired workers, outsourced personnel, and those suffering from tumors, fracture, chronic infection or systemic and neurologic diseases were excluded. The present study protocol was approved by the Institutional Review Board, Royal Thai Army Medical Department.

### Outcome

The outcome in the present study was work ability assessed by the work ability index (WAI). This questionnaire was developed by the Finnish Institute of Occupational Health in the 1980s comprising seven items including current work ability compared with the lifetime best, work ability in relation to the demands of the job, number of current diseases diagnosed by a physician, estimated work impairment due to diseases, sick leave during the past year (12 months), own prognosis of work ability 2 years from now, and mental resources [15]. All items were weighted and summed up to a total score of seven (the poorest) to 49 (the best). The present study used a short version with 14 groups of diseases in the third item (51 diseases in the original version) [16]. In the present study, the work ability index was dichotomized as good (37 – 49) and poor (7 – 36) [17].

### Exposure

The exposure in the present study was the number of pain sites assessed by self-administered paper questionnaire with a question on pain symptom in four-different anatomical regions including neck, upper extremities, low back, and lower extremities within the last month. Pain at the upper and lower extremities was recorded including the right, left, or both sides [12]. Pain intensity of each region was measured by zero (no pain) to ten (the worst pain) on a numerical pain rating scale. Each answer was dichotomized according to a numerical pain rating scale: less than five (no) and at least five (yes). Binary outcome of four-different anatomical regions was computed as the number of pain sites and categorized in three groups including no pain (0), few pain sites (1 – 2 sites), and many pain sites (3 – 4 sites).

### Confounders

Age, stress problem, and satisfaction in workload, payment, rest time, teamwork, and working environment were included in multivariable logistic regression analysis as confounders of the association between the number of pain sites and poor work ability [9, 18–21]. Age was categorized in two groups: less than 40 years and at least 40 years old. Stress problem was evaluated by a stress test (ST5) questionnarie which was developed by Department of Mental Health, Ministry of Public Health of Thailand comprising five items: sleep problem, decreased concentration, irritability, boredom, and social isolation. Each item was scored between zero and three, so its total score of ST5 questionnaire ranged from 0 to 15. Scores were dichotomized into categorical data of stress problem (No = not more than four, and Yes = more than four) [22]. Each topic of job satisfaction was measured by a 5-level Likert scale: highly satisfied, satisfied, partially satisfied, not satisfied, or not at all satisfied. However, in statistical analysis, these data were divided into two categories of satisfaction (Satisfied = highly satisfied or satisfied, and Not satisfied = partially satisfied, not satisfied, or not at all satisfied).

### Statistical analysis

The sample size was calculated based on the results of the study conducted by Neupane *et al.* [13]. Power of 80 % was selected for the Chi-square test. To detect computed effect size of 0.054 with an alpha level of 0.05, a total required sample of 222 participants was expected. Assuming a 70 % rate of response, a total of 318 participants would be required.

Logistic regression was conducted to determine the number of pain sites associated with poor work ability. The association was presented by odds ratio and its 95 % confident interval. Dummy variables were used to put number of pain sites (no pain = reference group) in the model. As potential confounders, sex (0 = male, 1 = female), age (0 = <40 years, 1 = ≥40), stress problem (No = 0, Yes = 1), satisfaction in workload (Satisfied = 0, Not satisfied = 1), satisfaction in payment (Satisfied = 0, Not satisfied = 1), satisfaction in rest time (Satisfied = 0, Not satisfied = 1), satisfaction in teamwork (Satisfied = 0, Not satisfied = 1), and satisfaction in working environment (Satisfied = 0, Not satisfied = 1) were included in the logistic regression analysis. In addition, before conducting the regression model, all potential confounders were tested by a formal-detection tolerance for multicollinearity diagnostics. A tolerance of less than 0.2 indicates a multicollinearity problem. All statistical analyses were performed using STATA version 10.

## Results

### Participant characteristics

A total of 254 health care providers participated in the study and completed all questionnaires (response rate of 79.9 %). According to the demographic characteristics (Table 1), the majority of participants were female (73.2 %) with a mean age of 33.9 (9.5) years ranging from 18 to 58 years. Of these, 28.7 %, 23.2 %, 20.5 %, 11.8 %, 10.6 %, and 5.1 % were nurses, physicians, pharmacists, medical technician, dentist, and physical/occupational therapist, respectively.

**Table 1:** Demographic characteristics of the participants

Variable	N	%
Age (year) – mean (SD)		33.9 (9.5)
Female	186	73.2
BMI		
<18.5 kg/m^2^	20	7.9
18.5 – 24.99 kg/m^2^	165	65.2
≥25 kg/m^2^	68	26.9
Occupation		
Physician	59	23.2
Pharmacist	52	20.5
Nurse	73	28.7
Dentist	27	10.6
Medical technician	30	11.8
Physical and occupational therapist	13	5.1
No stress problem	122	48.0
Satisfied in workload	148	58.3
Satisfied in payment	76	29.9
Satisfied in rest time	109	42.9
Satisfied in teamwork	165	65.0
Satisfied in working environment	141	55.5
Neck pain	63	24.8
Upper extremities pain	45	17.8
Low back pain	67	26.6
Lower extremities pain	72	28.3
Number of pain sites		
No pain	136	53.5
Few pain sites	79	31.1
Many pain sites	39	15.4

### Musculoskeletal pain and work ability

Among 254 participants, poor work ability was reported by 65 participants (25.6 %). The prevalence of any musculoskeletal pain during the past month was 46.5 %. Pain in the lower extremities was most frequently reported (28.3 %), followed by low back pain (26.6 %), neck pain (24.8 %) and upper extremities pain (17.8 %). In all, 31.1 % of participants had few pain sites (1 – 2 sites), whereas 15.4 % had many pain sites (3 – 4 sites).

### Impact of the number of pain sites on work ability

As shown in Table 2, the prevalence of poor work ability in participants who had no pain, few pain sites, and many pain sites were 18.4 %, 30.4 %, and 41.0 %, respectively. In crude analysis, a statistically significant association was found between the number of pain sites and work ability. Odds ratio of poor work ability in participants who had few pain sites was 1.9 (95 % CI 1.01 – 3.69), while in those who had many pain sites was 3.0 (95 % CI 1.42 – 6.68).

**Table 2:** Odds ratio of poor work ability from multivariable logistic regression analysis

	All subjects	No. of subjects with poor work ability (%)	Crude OR (95 % CI)	Adjusted OR^a^ (95 % CI)
No pain	136	25 (18.4)	1	1
Few pain sites	79	24 (30.4)	1.9 (1.01 – 3.69)**	1.85 (0.91 – 3.76)
Many pain sites	39	16 (41.0)	3.0 (1.42 – 6.68)**	2.41 (1.04 – 5.58)**

^a^Adjusted for sex, age group, stress, satisfaction in workload, satisfaction in payment, satisfaction in rest time, satisfaction in teamwork, satisfaction in working environment

**P value <0.05

To eliminate the effect of confounders, logistic regression analysis was conducted. All of the potential confounders were tested for multicollinearity diagnostics. A formal-detection tolerance of all of these ranged from 0.6 to 0.9, so there was no multicollinearity problem. After adjustment for many confounders, odds ratio in those who had few pain sites showed no statistical significance (1.89 (95 % CI 0.93 – 3.80)), whereas those who had many pain sites still showed statistical significance although its odds ratio showed a small decrease (2.44 (95 % CI 1.06 – 5.66)).

## Discussion

The present study is the first to reveal the negative impact of the number of pain sites on work ability among health care providers. The hypothesis in this study was the association between musculoskeletal pain and poor work ability would be stronger when the number of pain sites increased. The main findings supported this hypothesis.

The main results showed that participants who had few pain sites were 1.9 time more likely to develop poor work ability than those who had none, while this probability increased to 3.0 times in participants who had many pain sites by crude analysis. After adjustment for age, stress problem, and job sattisfaction in the regression model, the tendency for this result still remained. Hence, the number of pain sites could predict the probabity of poor work ability among health care providers. However, the results in those who had few pain sites were not significant by multivariable analysis. This was likely caused by the small sample size resulting in a wide 95 % confident interval. To compare with previous studies, these findings were similar to the health survey among Finnish general workers, and the prospective study conducted among Finnish industrial workers [12, 13].

The present study had several stengths. Firstly, this study focused on musculoskeletal pain within the last month to reduce the recall bias. Next, the WAI, one of the the validated international questionnaires of work ability assessment, was used to determine poor work ability [15]. Importantly, this study was concerned with the effect of some potential confounders and included them in the logistic regression model.

On the other hand, some weaknesses were observed in this study. The major limitation was the lack of a temporal relationship between the number of pain sites (exposure) and work ability (outcome) because this study employed a cross-sectional design. In addition, This study was carried out only in a tertiary care center involving a great workload and number of responsibilities, so the results from this study could not be applied to other hospital care settings such as a primary care unit. Moreover, all data were collected by the self-administered questionnaire without objective assessment, for example, a physical examination to determine musculoskeletal pain. Hence, information bias or invalidation of data might occur, but a questionnaire survey seem to be the practical way to assess musculoskeletal pain in epidemiological studies [13]. Finally, Participants who had many pain sites tended to have chronic pain which might have affected work ability [3]. Therefore, a subgroup analysis divided by pain duration could be conducted in further studies.

## Conclusions

The probability of poor work ability would be higher when the number of pain sites increased. Multi-site musculoskeletal pain should be concerned not only in industrial working populations but also in hospital working populations, and should be specially attended in occupational health services, for example, annual health examination, ergonomic concerns, and walk-through survey. In addition, among health care providers, the self-administered questionnaire for simply counting the number of pain sites could be a screening instrument to detect workers who have the risk of poor work ability.

## Abbreviations

DALYs: Disability-adjusted life-years
WAI: Work ability index
